# Editorial: Gastrointestinal Tumor Heterogeneity and Related Anti-Cancer Strategies

**DOI:** 10.3389/fonc.2022.873240

**Published:** 2022-04-11

**Authors:** Rui Liao, Yu-Jun Shi, Michael D. Chuong, Ju Cao

**Affiliations:** ^1^ Department of Hepatobiliary Surgery, The First Affiliated Hospital of Chongqing Medical University, Chongqing, China; ^2^ Institute of Clinical Pathology, Key Laboratory of Transplant Engineering and Immunology, National Health Commission (NHC), West China Hospital, Sichuan University, Chengdu, China; ^3^ Department of Radiation Oncology, Miami Cancer Institute, Baptist Health South Florida, Miami, FL, United States; ^4^ Herbert Wertheim College of Medicine, Florida International University, Miami, FL, United States; ^5^ Department of Laboratory Medicine, The First Affiliated Hospital of Chongqing Medical University, Chongqing, China

**Keywords:** gastrointestinal tumor, tumor microenvironment, inter-patient heterogeneity, intra-tumor heterogeneity, phenotypic plasticity, targeted therapy

The significance of tumor microenvironment (TME) heterogeneity is increasingly becoming recognized as playing an essential role in tumorigenesis and malignant biological behaviors. Dr. Hanahan D (1) presents several prospective new cancer hallmarks and its enabling characteristics closely associated with tumor heterogeneity: 1) unlocking phenotypic plasticity, 2) non-mutational epigenetic reprogramming, 3) polymorphic microbiomes, and 4) senescent cells. The new facets of the conceptualization of cancer have a heuristic value in paving the way for the development of precision therapies and new targeted therapies. A growing knowledge base has revealed that the heterogeneity of gastrointestinal tumors, including inter-patient heterogeneity (IPH) and intra-tumor heterogeneity (ITH) of gastric cancer, colorectal cancer, hepatocellular carcinoma (HCC), cholangiocarcinoma, pancreatic cancer, gallbladder carcinoma, and esophageal adenocarcinoma, is a determining factor of tumor development (2, 3). Therefore, based on molecular heterogeneity in TME, more novel and effective anti-cancer therapeutic algorithms have been discussed to selected subsets of gastrointestinal tumors.

Of note, inter-patient molecular heterogeneity has hampered the clinical practice of an expanding variety of targeted therapies and personalizing their prescriptions. An inter-patient molecular heterogeneity investigation using genomic and transcriptomic data for 4890 tumors from The Cancer Genome Atlas database showed that the repertoires of molecular targets of the clinical recommendations for accepted drugs were not congruent with the gene mutation patterns of different cancer types (4). Due to IPH, gastrointestinal tumors between individual patients frequently exhibit distinct clinical behaviors and treatment response produced by high levels of transcriptomic and (epi)genomic variation. A comprehensive single-cell profile of gastric cancer across clinical stages and histological subtypes identified 34 distinct cell-lineage states, and highlighted inter- and intra-lineage similarities and differences between patient-derived organoids and primary tumors (5). Enhancer variation has been identified as a major cause of IPH in cancer. Histone modification and functional assay data may be one of the options contributing genetic (e.g. ING1, ARL4C) and regulatory trans-acting factor (e.g. HNF4α) mechanisms to gastric cancer enhancer functional heterogeneity (6).

Also, ITH poses an important clinical challenge in therapeutic resistance. To better address the origin and drivers of ITH across cancer types, a robust consensus strategy has been developed to assess ITH and its origin, drivers, and recurrent changes in mutation signature activity *via* copy number and cluster mutations (7). This study underlined the importance of ITH and also provided detailed insight into tumor evolutionary dynamics. In HCC, the heterogeneous genomic landscape may facilitate effective anti-cancer therapeutic algorithms of personalized management. For instance, the molecular profile of the original tumor revealed that patients with intra-hepatic metastases should receive targeted therapy, whereas patients with multicentric tumors patients sharing the same genetic and environmental backgrounds could benefit from treating the underlying liver disease (8). An atlas of inter- and intra-tumor heterogeneity of apoptosis competency in colorectal cancer suggested that ITH may represent an intrinsic, non-genomic property instead of increase with the process of malignant transformation (9).

Therefore, further in-depth analyses of IPH and ITH in larger patient cohorts with gastrointestinal tumors are required. This Research Topic collection embodies 16 multidisciplinary articles focused on “gastrointestinal tumor heterogeneity and related anti-cancer strategies”. Overall, the 16 papers in this Research Topic discussed gastrointestinal IPH and ITH deepening mechanistic insights, involved with basic experimental research, and clinical outcome predictive model and bioinformatics analysis for early diagnosis and targeted therapies of tumors. Specifically in this Research Topic, Kim et al. reported that dynamic changes in serum KRAS^G12/13^ mutation heterogeneous status in serum cell-free DNA represented a potential source for monitoring recurrence of colorectal adenocarcinoma. Moreover, Gao et al. found the HCC patients from distinct immune subclasses with various heterogeneous statuses had different clinical prognoses and responses to personalized treatment through tumor transcriptome data analysis. In a review article, Li et al. summarized current advances concerning the reciprocal crosstalk of malignant cells and mesenchymal stem cells in the progression of gastric cancer, stressed the complexity and heterogeneity of tumor-stroma connections, and discussed their underlying therapeutic implications.

We sincerely thank all the authors, various reviewers/editors of the respective manuscripts, and the editorial team at Frontiers for their assistance and support in the process of reviewing and publishing this Research Topic, “Gastrointestinal Tumor Heterogeneity and Related Anti-Cancer Strategies”. The next decade promises to illuminate more in-depth aspects of tumor heterogeneity contributing to gastrointestinal cancer prevention and treatments.

## Author Contributions

RL wrote this manuscript. YJ, MC, and JC reviewed it. All authors listed have made a direct and intellectual contribution to the work, and approved the submitted version.

## Funding

This research was supported by the National Natural Science Foundation of China (No. 82072689), Science and Health Joint Research Project of Chongqing Municipality (2020GDRC013).

## Conflict of Interest

The authors declare that the research was conducted in the absence of any commercial or financial relationships that could be construed as a potential conflict of interest.

## Publisher’s Note

All claims expressed in this article are solely those of the authors and do not necessarily represent those of their affiliated organizations, or those of the publisher, the editors and the reviewers. Any product that may be evaluated in this article, or claim that may be made by its manufacturer, is not guaranteed or endorsed by the publisher.
